# Supplementary figures and images for: Co-expression clustering across flower development identifies modules for diverse floral forms in Achimenes (Gesneriaceae)
Source: PeerJ. 2020 Mar 11;8:e8778. doi: 10.7717/peerj.8778 (PMC7071821; doi:10.7717/peerj.8778)

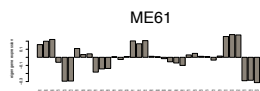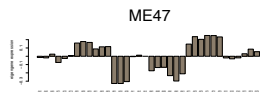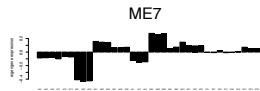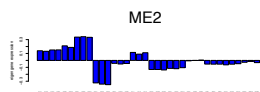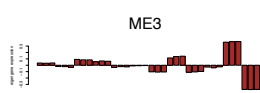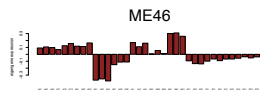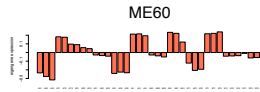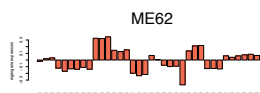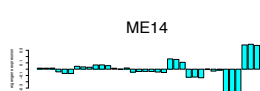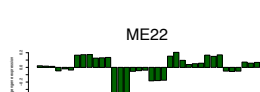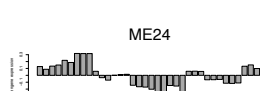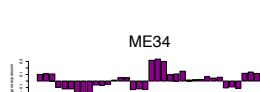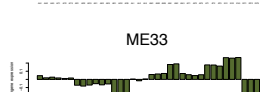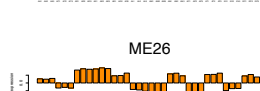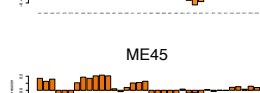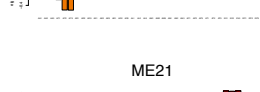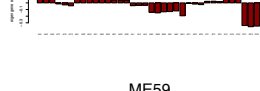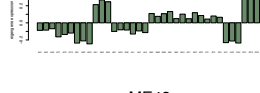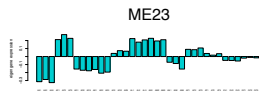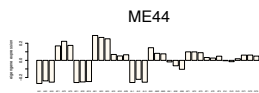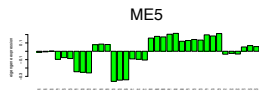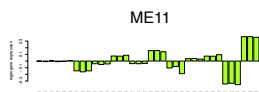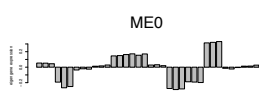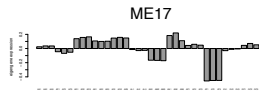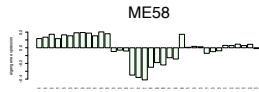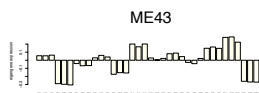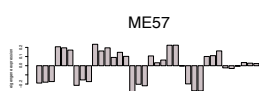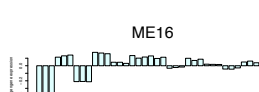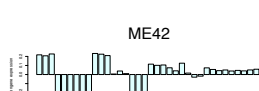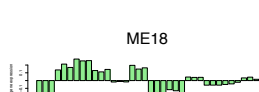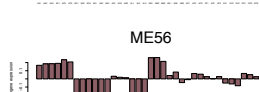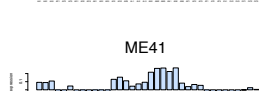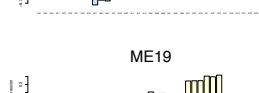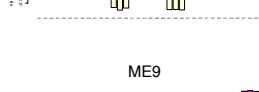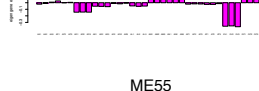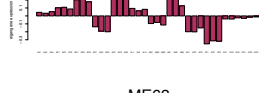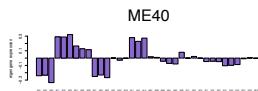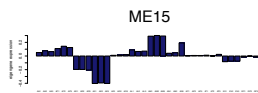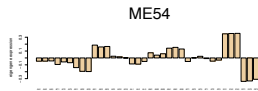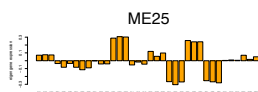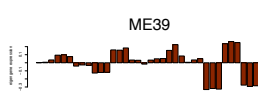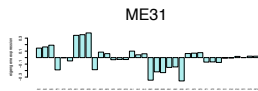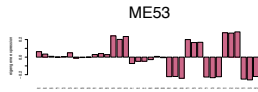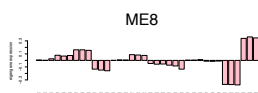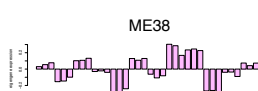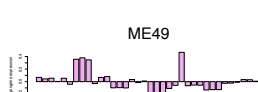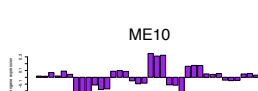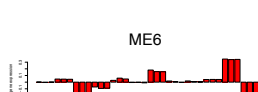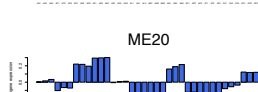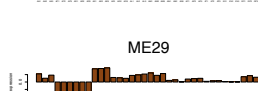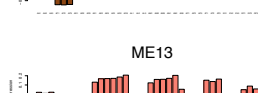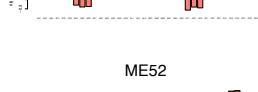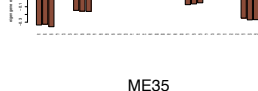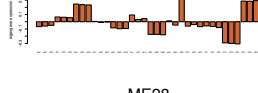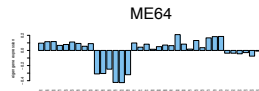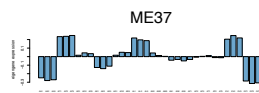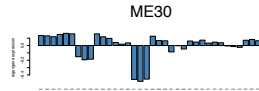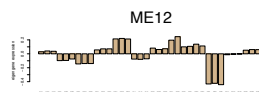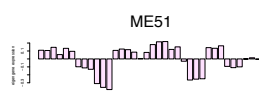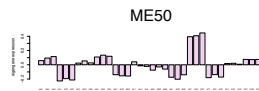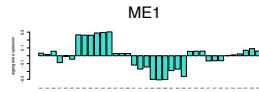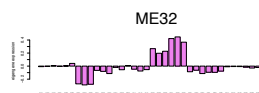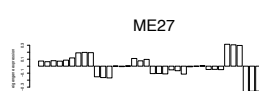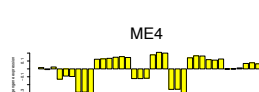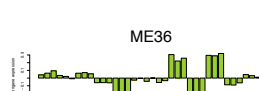

Supplement: Supplemental Information 2 — Plots show the module eigengenes for each module in the Bud stage network. Each bar represents the eigengene expression (y-axis) for each sample in the Bud stage dataset (x-axis). Eigengene expression represents the expression of a sample within a module. [file peerj-08-8778-s002.pdf]

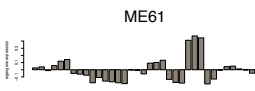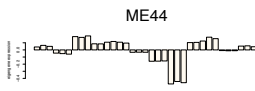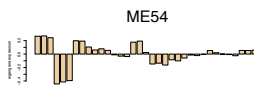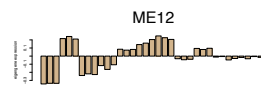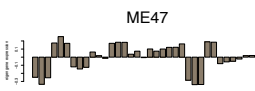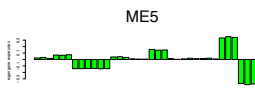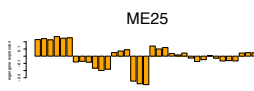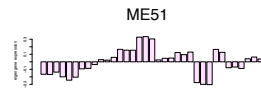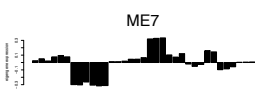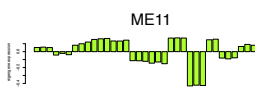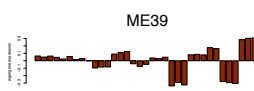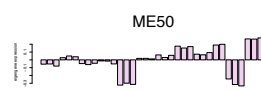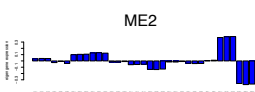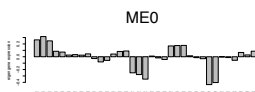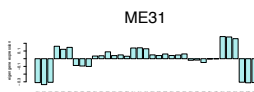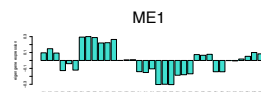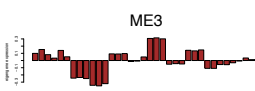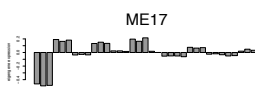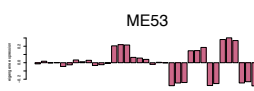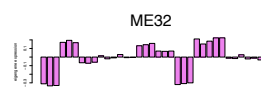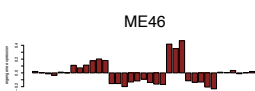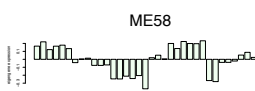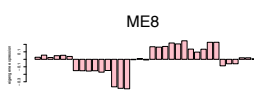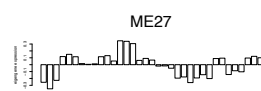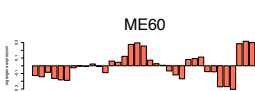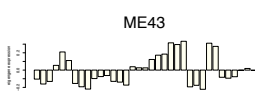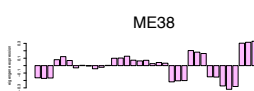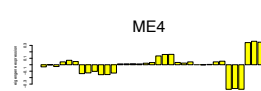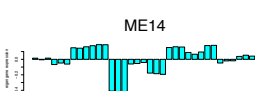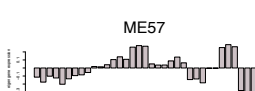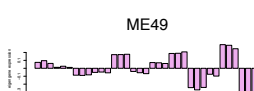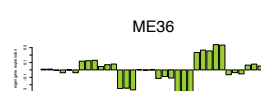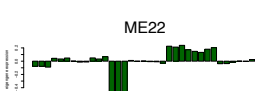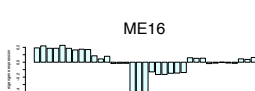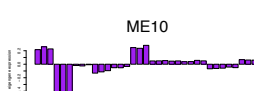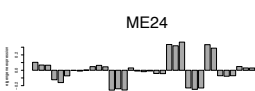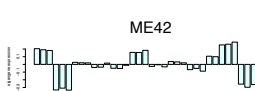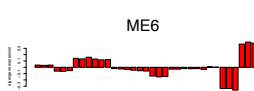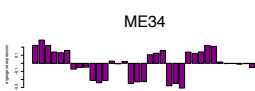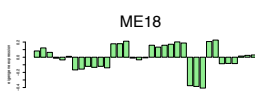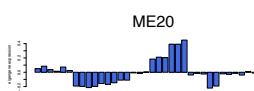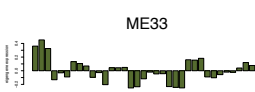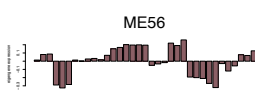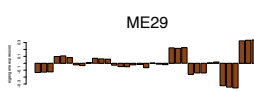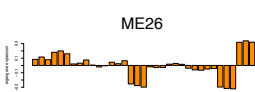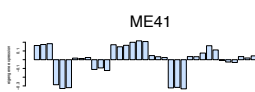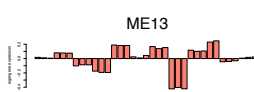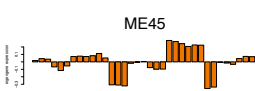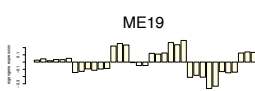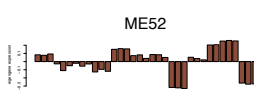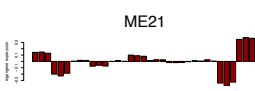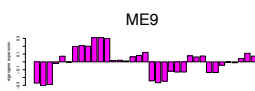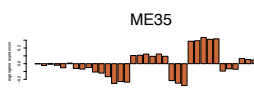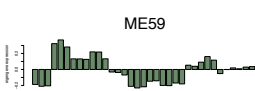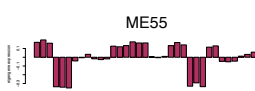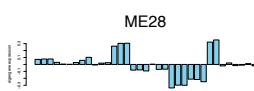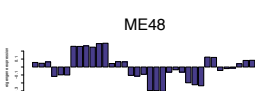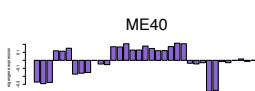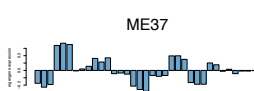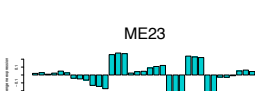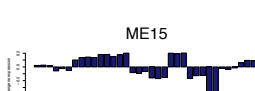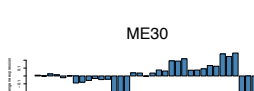

Supplement: Supplemental Information 3 — Plots show the module eigengenes for each module in the D stage network. Each bar represents the eigengene expression (y-axis) for each sample in the D stage dataset (x-axis). Eigengene expression represents the expression of a sample within a module. [file peerj-08-8778-s003.pdf]

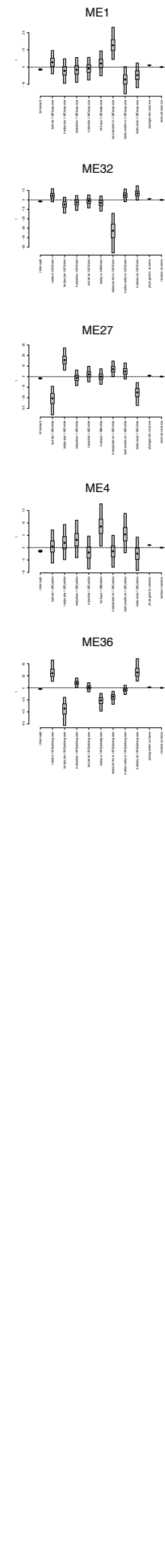

Supplement: Supplemental Information 4 — Each plot shows a summary of the important floral predictors (flower color, flower shape, and corolla spur) for each module in the Bud stage network. The posterior distribution of an independent variable with negligible effect on eigengene expression is centered around zero (dashed line). The distribution of an influential variable is expected to be substantially different from zero. Black dots indicate the mode of the posterior distribution, and boxes indicate the 50 and 95% credible intervals. [file peerj-08-8778-s004.pdf]

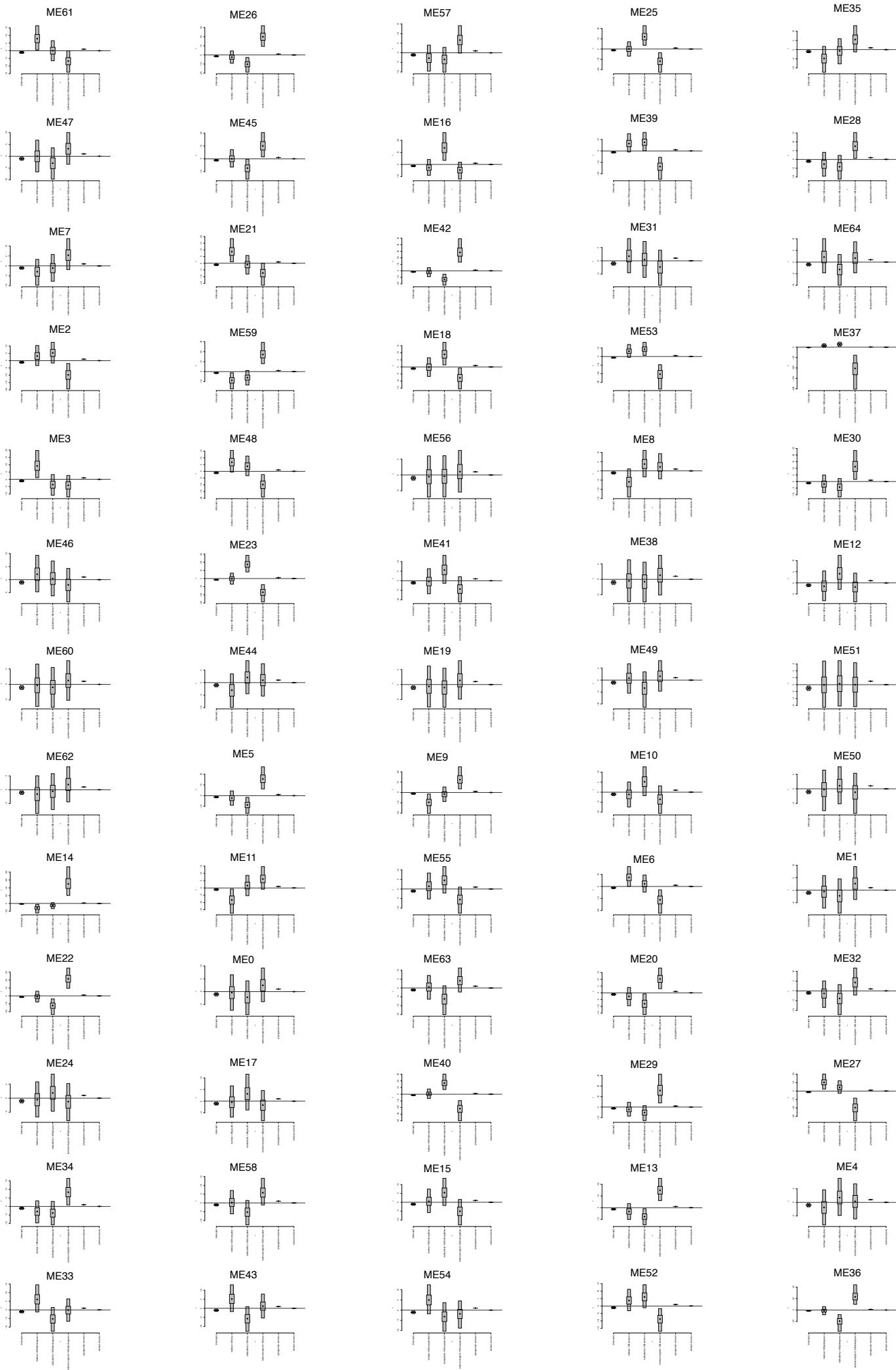

Supplement: Supplemental Information 5 — Each plot shows a summary of the important pollination syndrome predictors for each module in the Bud stage network. The posterior distribution of an independent variable with negligible effect on eigengene expression is centered around zero (dashed line). The distribution of an influential variable is expected to be substantially different from zero. Black dots indicate the mode of the posterior distribution, and boxes indicate the 50 and 95% credible intervals. [file peerj-08-8778-s005.pdf]

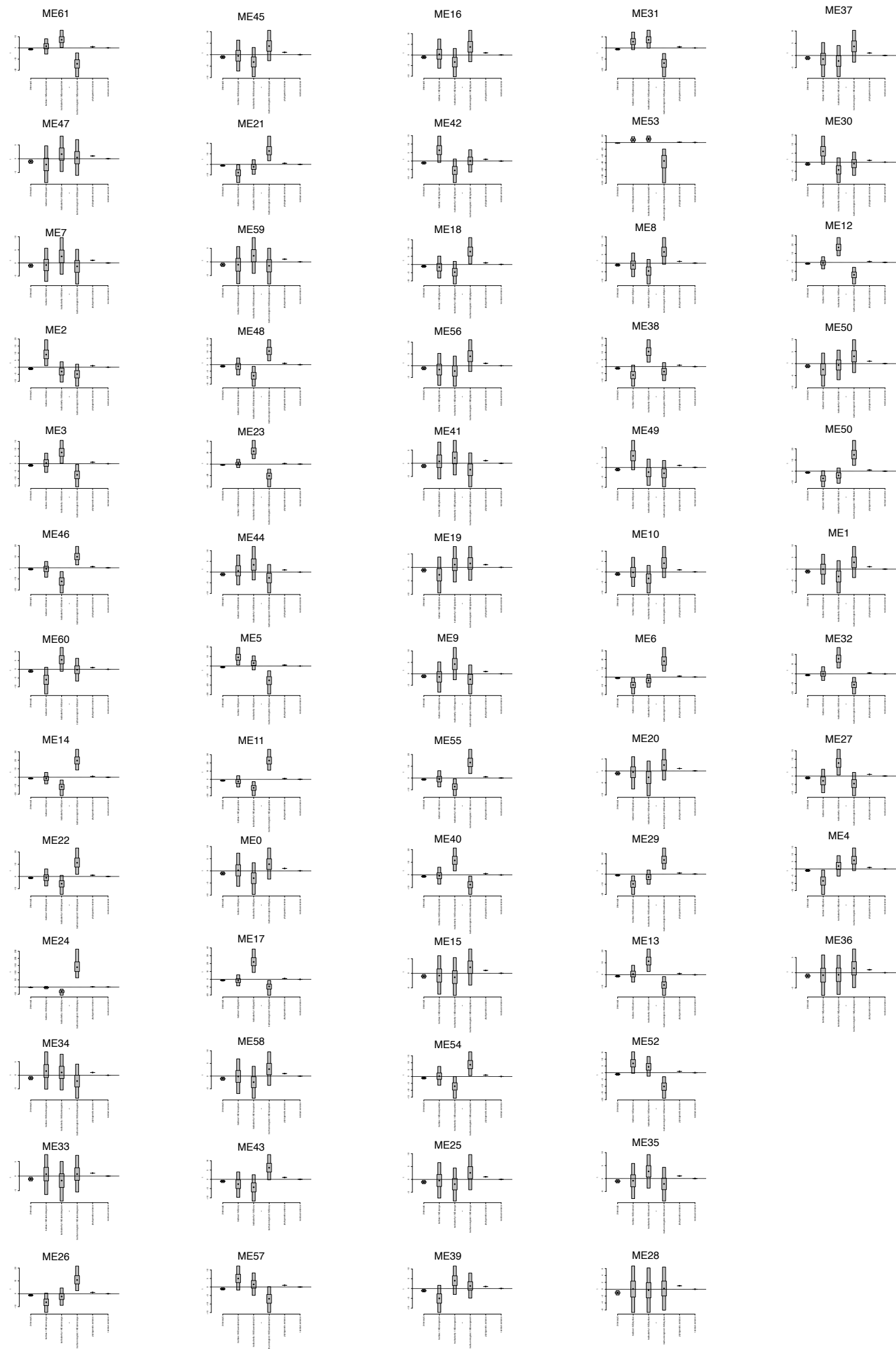

Supplement: Supplemental Information 7 — Each plot shows a summary of the important pollination syndrome predictors for each module in the D stage network. The posterior distribution of an independent variable with negligible effect on eigengene expression is centered around zero (dashed line). The distribution of an influential variable is expected to be substantially different from zero. Black dots indicate the mode of the posterior distribution, and boxes indicate the 50 and 95% credible intervals. [file peerj-08-8778-s007.pdf]

**A****Bud stage**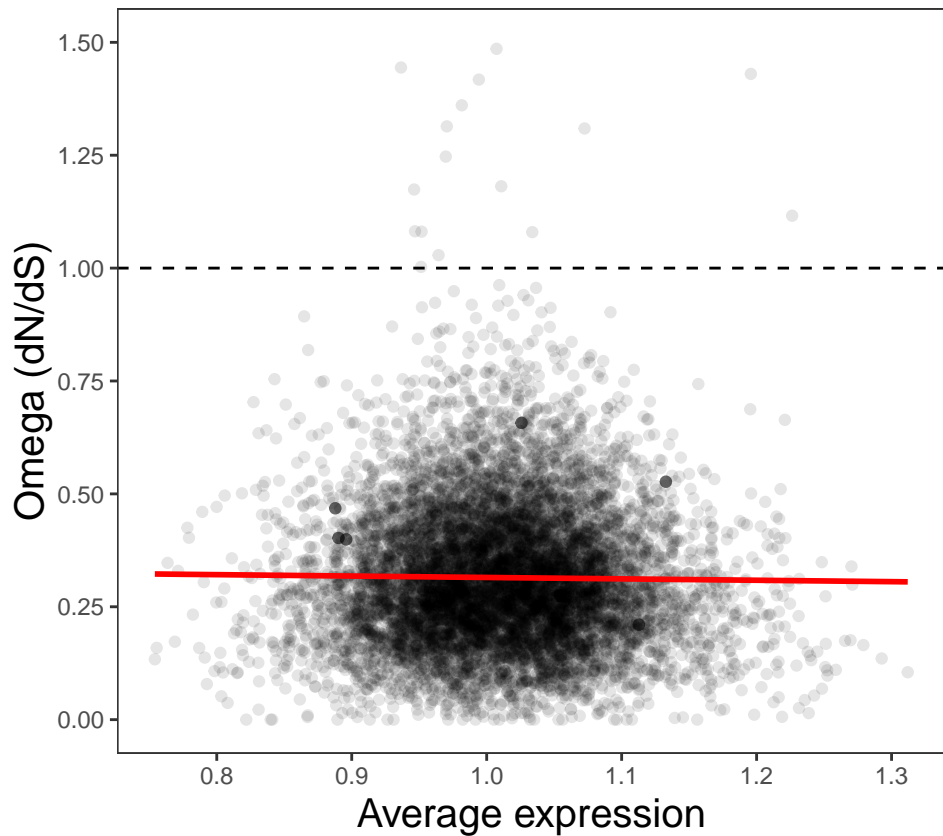**B****D stage**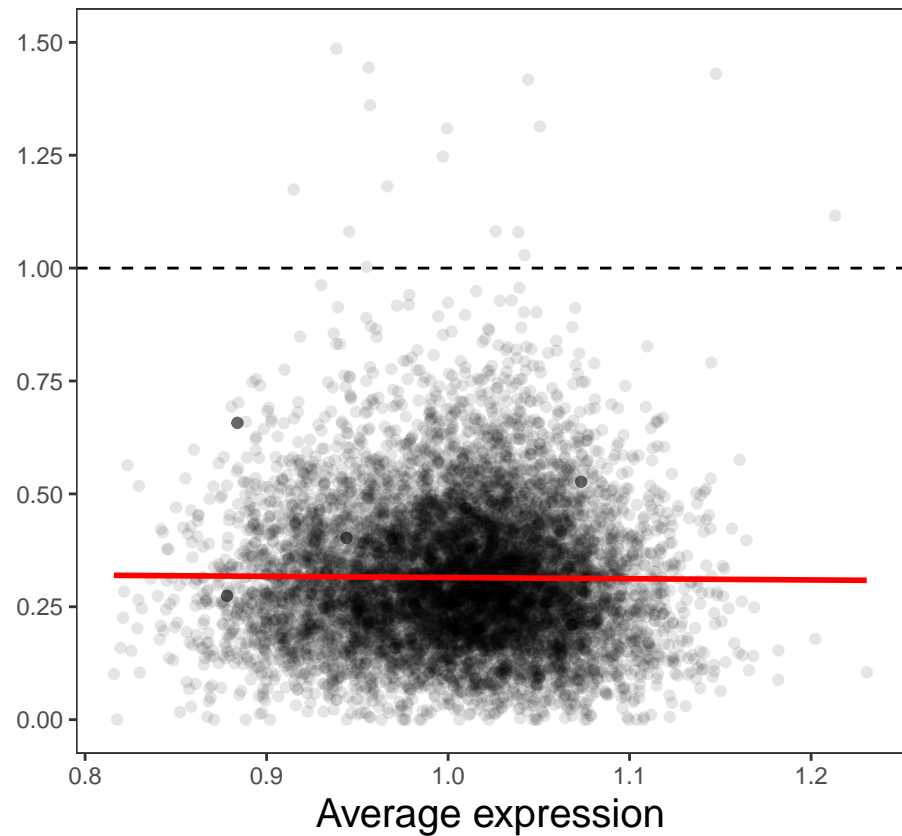

Supplement: Supplemental Information 8 — (A) Bud stage. (B) D stage. Each point indicates an individual orthogroup. The red line indicates the linear regression line between fit for dN/dS ~ expression. The dotted line indicates dN/dS = 1, where orthogroups with dN/dS > 1 may be under stronger relaxed selection. [file peerj-08-8778-s008.pdf]

**A****Bud stage**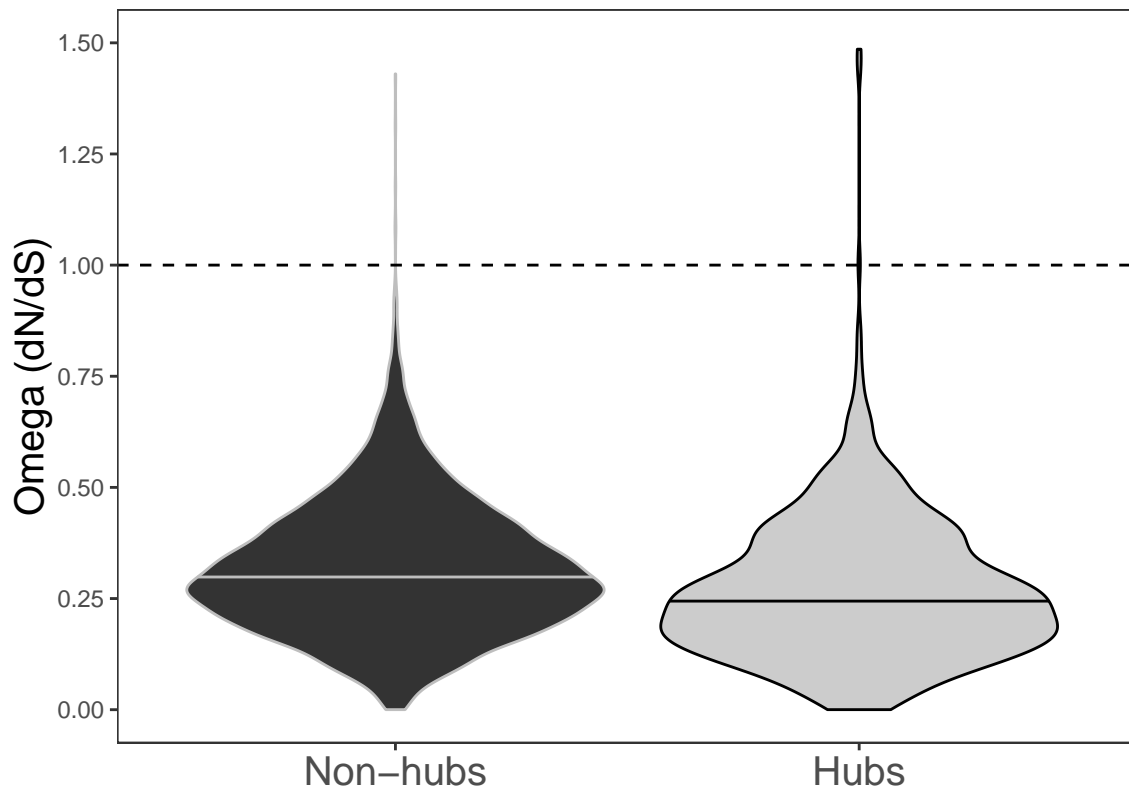**B****D stage**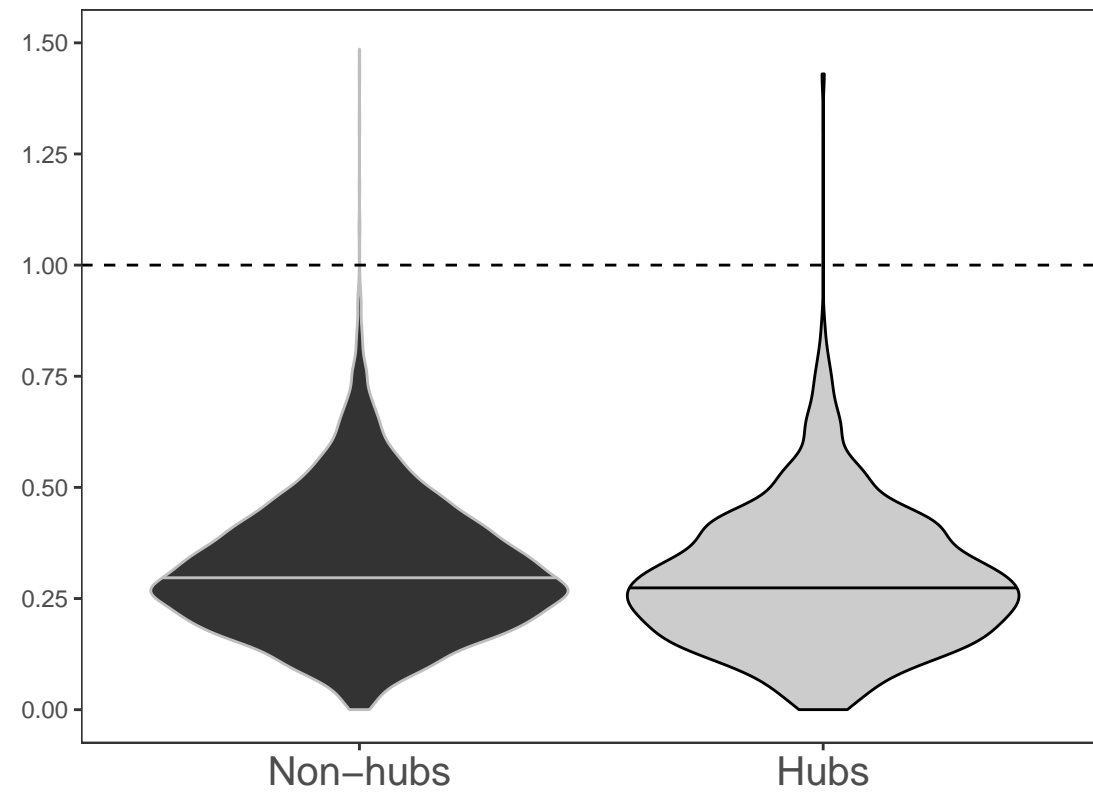**C****Bud stage**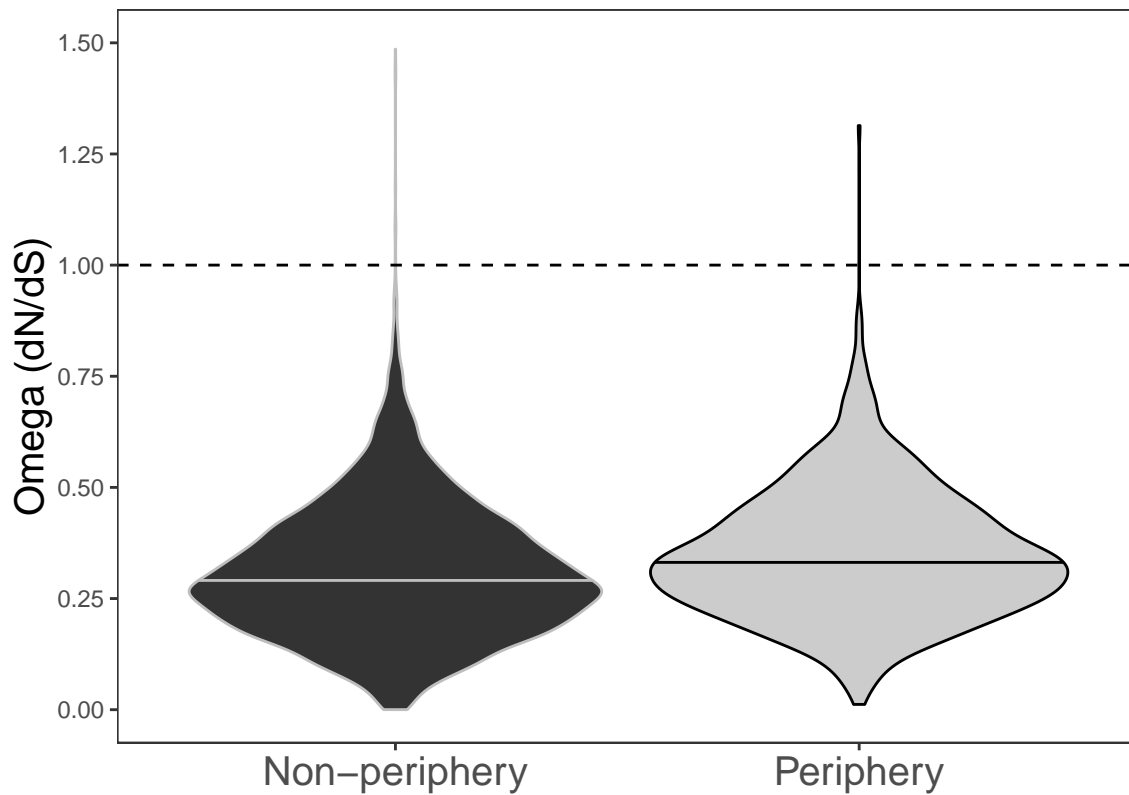**D****D stage**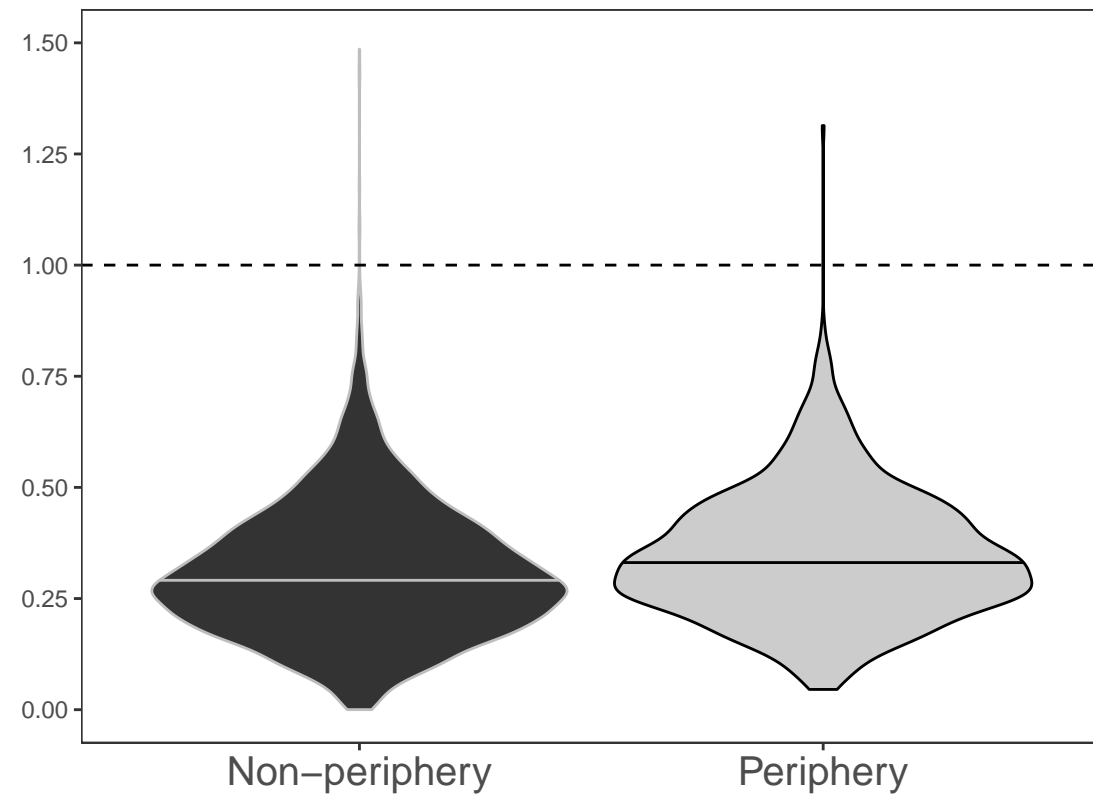

Supplement: Supplemental Information 9 — (A) dN/dS for hub and non-hub nodes in the Bud stage network. (B) dN/dS for hub and non-hub nodes in the D stage network. (C) dN/dS for periphery and non-periphery nodes in the Bud stage network. (D) dN/dS for periphery and non-periphery nodes in the D stage network. Violin plots show the distribution of dN/dS values for each category. Horizontal lines in each distribution indicate the median dN/dS value. [file peerj-08-8778-s009.pdf]
