# Supplementary material for: Co-expression clustering across flower development identifies modules for diverse floral forms in Achimenes (Gesneriaceae)
Source: PeerJ. 2020 Mar 11;8:e8778. doi: 10.7717/peerj.8778 (PMC7071821; doi:10.7717/peerj.8778)
Supplement: Supplemental Information 6 — Each plot shows a summary of the important floral predictors (flower color, flower shape, and corolla spur) for each module in the D stage network. The posterior distribution of an independent variable with negligible effect on eigengene expression is centered around zero (dashed line). The distribution of an influential variable is expected to be substantially different from zero. Black dots indicate the mode of the posterior distribution, and boxes indicate the 50 and 95% credible intervals. [file peerj-08-8778-s006.pdf]

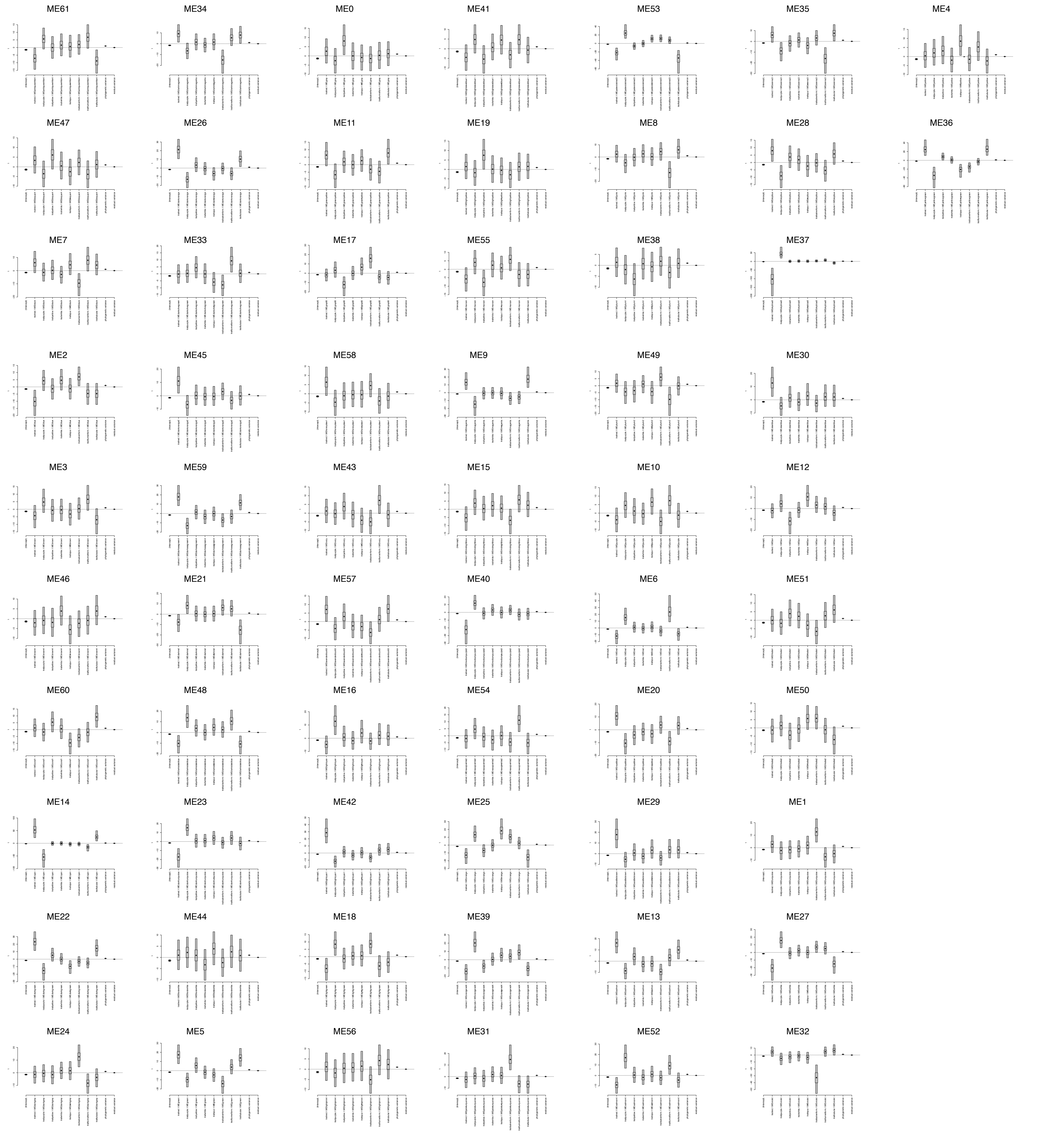
ME61
ME34
ME0
ME41
ME53
ME35
ME4
ME47
ME26
ME11
ME19
ME8
ME28
ME36
ME7
ME33
ME17
ME55
ME38
ME37
ME2
ME45
ME58
ME9
ME49
ME30
ME3
ME59
ME43
ME15
ME10
ME12
ME46
ME21
ME57
ME40
ME6
ME51
ME60
ME48
ME16
ME54
ME20
ME50
ME14
ME23
ME42
ME25
ME29
ME1
ME22
ME44
ME18
ME39
ME13
ME27
ME24
ME5
ME56
ME31
ME52
ME32
